# Effects of a cognitive-poetics-based narrative intervention using British and American coming-of-age novels on children's executive function and psychological adjustment: a randomized controlled study

**DOI:** 10.3389/fpsyg.2026.1952178

**Published:** 2026-10-26

**Authors:** Shanshan Li, Shuwei Xie

**Affiliations:** 1 School of Chinese Language and Literature, Xinjiang University, Urumqi, Xinjiang, China; 2 Department of Dance, College of Performing Arts & Sport, Hanyang University, Seoul, Republic of Korea

**Keywords:** children, cognitive poetics, cognitive reappraisal, coming-of-age fiction, executive function, narrative intervention, narrative transportation, psychological adjustment

## Abstract

**Introduction:**

Children aged 10–12 years are moving through late childhood toward early adolescence, when executive control, emotion regulation, and social understanding are still developing. Literature-based activities offer one setting in which children can consider other perspectives and reinterpret difficult events, yet cognitive-poetics-informed interventions have rarely been tested with rigorous longitudinal designs. We examined whether a structured cognitive-poetics-based narrative intervention using British and American coming-of-age fiction was associated with changes in executive function and psychological adjustment. Narrative transportation and cognitive reappraisal were examined as candidate mechanisms.

**Methods:**

In a 12-week parallel-group randomized study, 180 children aged 10–12 years were allocated 1:1 to a cognitive-poetics-based narrative intervention (*n* = 90) or an active literary-reading control (*n* = 90). Both conditions involved three 40-min sessions per week. Assessments were conducted at baseline (T0), week 6 (T1), week 12 (T2), and a 4-week post-intervention follow-up (T3). Executive function was represented by inhibitory-control, working-memory, and task-switching performance; psychological adjustment was assessed using the RCADS, SDQ, and cognitive-reappraisal measures. Longitudinal effects were tested with REML linear mixed-effects models using participant random intercepts and an AR(1) residual structure. A temporally ordered indirect-effect model tested intervention assignment → T1 narrative transportation → T2 cognitive reappraisal → T3 outcomes, controlling baseline reappraisal and the corresponding baseline outcome.

**Results:**

Significant group × time interactions were observed for the executive-function composite (F = 18.64, *p* < 0.001, partial η^2^ = 0.095), RCADS total score (F = 15.72, *p* < 0.001, partial η^2^ = 0.086), and SDQ difficulties score (F = 12.48, *p* < 0.001, partial η^2^ = 0.074). At T2, the displayed summary statistics correspond to unadjusted between-group standardized mean differences of d = 0.38 (95% CI [0.07, 0.68]) for executive function, d = −0.82 (95% CI [−1.14, −0.51]) for RCADS, d = −0.65 (95% CI [−0.96, −0.34]) for SDQ difficulties, and d = 0.81 (95% CI [0.50, 1.12]) for cognitive reappraisal. In the temporally ordered path model, the standardized serial-specific indirect association was 0.04 for executive function and −0.05 for psychological difficulties; when parallel indirect routes through transportation and reappraisal were included, the corresponding total indirect effects were 0.14 (95% CI [0.06, 0.25]) and −0.18 (95% CI [−0.31, −0.09]), respectively. A structured cognitive-poetics-based narrative intervention was associated with better executive-function performance and several non-clinical psychological-adjustment indicators than an active reading control. Some group differences remained visible 4 weeks later, although the follow-up was too short to support claims of long-term maintenance. The pattern warrants further study of how guided literary processing may contribute to children's cognitive and emotional development.

## 1 Introduction

### 1.1 Developmental context: executive function and psychological adjustment at ages 10–12

At ages 10–12, executive abilities that support schoolwork, goal-directed behavior, emotion regulation, and social adaptation are still maturing (Best et al., 2009; Diamond, 2013). It is useful to separate these abilities rather than treat executive function as one undifferentiated skill. Inhibitory control helps suppress dominant or distracting responses; working memory keeps task-relevant information active and updates it; cognitive flexibility supports switching between rules, representations, or perspectives (Miyake et al., 2000; Diamond, 2013). The three processes are related, but they do not necessarily develop in parallel, and classroom activities may draw on them in different combinations.

Psychological adjustment is changing during the same period as children negotiate school demands, peer relationships, family life, and a growing capacity for self-evaluation. Universal school-based programs can improve social-emotional outcomes, but the size and durability of those effects vary with program content, implementation, participant characteristics, and the way outcomes are defined (Durlak et al., 2011; Taylor et al., 2017). This is one reason ordinary educational activities that engage cognitive control and emotion regulation are worth examining without framing participating children as psychiatric patients.

### 1.2 Narrative approaches, bibliotherapy, and coming-of-age fiction

Narrative and literature-based approaches overlap in some respects, but they are not the same intervention. Clinical narrative therapy comes from systemic and postmodern psychotherapy and works with personal problem narratives and re-authoring processes (White and Epston, 1990). Developmental or creative bibliotherapy instead uses selected fictional or literary texts, often in educational settings, to support reflection, emotional understanding, and coping. School counselors have adapted narrative principles for children and adolescents, whereas school-based bibliotherapy usually relies on structured reading and discussion rather than psychotherapy itself (Beaudoin et al., 2016; Romagnolo and Ohrt, 2017; Veronese and Barola, 2018). Narrative-therapy programs have also been studied with school-age populations, although their target groups and clinical intensity vary considerably (Peker et al., 2026; Zarra-Nezhad et al., 2023).

In their 2026 review, Redman et al. (2026) examined 23 experimental or quasi-experimental studies of creative bibliotherapy delivered in schools to children aged 5–16 years. Results across that literature were encouraging in places but methodologically uneven. In studies reporting favorable outcomes, three implementation features appeared repeatedly: suitable text selection, structured interaction, and trained delivery. The same review also makes the methodological gap clear. School-based literary intervention is plausible, but stronger randomization, fuller intervention descriptions, and direct tests of proposed mechanisms are still needed.

We chose coming-of-age fiction for the situations it repeatedly places before young readers, not because we assumed the genre was uniquely effective. Young protagonists often face questions of autonomy, belonging, conflict, setbacks, identity, and changing relationships. These situations invite readers to reconsider how a character understood an event, what another person might have noticed, and how an interpretation changes when new information appears. Other genres can offer similar opportunities. Here, the narrower question was whether carefully selected coming-of-age texts could provide an age-appropriate setting for perspective shifting and cognitive reappraisal in children aged 10–12 years.

### 1.3 Cognitive poetics and candidate mechanisms

Cognitive poetics offers one way to describe what happens during this kind of reading. Readers build situation models or “text worlds,” distribute attention, mentally represent events, and connect textual information with prior knowledge and affective experience (Zwaan and Radvansky, 1998; Stockwell, 2002; Gavins and Steen, 2003). Following a developing narrative can require holding earlier events in mind, revising an interpretation when new information appears, and moving between character perspectives. Those operations resemble working-memory, inhibitory, and flexibility demands, although literary reading should not be treated as a substitute for direct executive-function training.

Narrative transportation was one candidate process. Green and Brock (2000) define transportation as cognitive, emotional, and imagery-based absorption in a narrative world. When a child becomes absorbed in a story, the character's goals and the consequences of events may carry more weight, while alternative interpretations can be considered at some distance from the child's own immediate experience. Fiction has also been linked with social-cognitive and empathic processes, although effect sizes and replicability have varied across paradigms (Mar et al., 2006; Bal and Veltkamp, 2013; Kidd and Castano, 2019).

Cognitive reappraisal was examined as a second candidate process. Reappraisal changes how a situation is interpreted and can thereby alter its emotional impact (Gross, 1998; Gross and John, 2003). Asking why a character acted in a particular way, or how the same setback might be understood differently, gives children repeated practice in generating another meaning. That makes reappraisal theoretically relevant here, but not a demonstrated mediator by itself. A stronger test requires temporal separation of mediator and outcome together with adjustment for their baseline levels.

### 1.4 Current study and hypotheses

We therefore compared a structured cognitive-poetics-based narrative intervention with an active literary-reading condition matched for reading material, contact frequency, and session duration. The contrast was designed to separate guided narrative processing from literary exposure itself. In other words, both groups read; only one group repeatedly practiced text-world construction, perspective shifts, alternative interpretation, and reappraisal.

**H1:** Children assigned to the cognitive-poetics-based narrative intervention would show greater improvement in executive-function performance than children assigned to the active reading control. **H2:** The intervention group would show greater reductions in anxiety/depressive symptoms and behavioral-emotional difficulties. **H3:** Narrative transportation would be positively associated with intervention-related changes. H4: Cognitive reappraisal would be associated with better psychological-adjustment outcomes and would contribute to the proposed indirect pathway. **H5:** Group differences observed at post-intervention would show short-term persistence at the 4-week follow-up. The 4-week assessment is treated as a short-term follow-up and not as evidence of long-term developmental maintenance.

## 2 Methods

### 2.1 Design, setting, ethics, and participants

This study used a two-arm, parallel-group randomized design with an active control condition. A total of 180 children aged 10–12 years were recruited through primary-school and community reading settings and randomized in a 1:1 ratio to the cognitive-poetics-based narrative intervention (*n* = 90) or active literary-reading control (*n* = 90). Assessments were scheduled at T0 (baseline), T1 (week 6), T2 (week 12/post-intervention), and T3 (4 weeks after intervention completion).

Participants were recruited through primary-school and community reading settings. Recruitment and assessment procedures were conducted according to the approved study protocol before randomization.

Before recruitment, the study protocol was reviewed and approved by the Korea-China Cultural and Arts Education Exchange Association (Approval No. HZWH-000101). Parents or legal guardians provided written informed consent, and participating children provided assent. Participation was voluntary, and children could discontinue without penalty. The intervention was educational and developmental, not a clinical treatment for a diagnosed psychiatric disorder.

Eligibility criteria were: (a) age 10–12 years; (b) ability to read and understand the age-appropriate study materials independently; (c) parental/guardian consent and child assent; and (d) completion of baseline assessment. Children were not enrolled if study records indicated severe reading difficulty that prevented participation in the reading tasks or a severe neurological, developmental, or psychiatric condition incompatible with the study procedures. Receipt of intensive psychological treatment during the intervention period was also an exclusion criterion in the original protocol.

Baseline reading ability was operationalized as a 0–100 curriculum-aligned reading-comprehension screening score administered before randomization. Children were required to score at least 60/100 and demonstrate independent comprehension of age-appropriate prose; the same baseline score was used as a stratification variable and is reported descriptively in Table 1.

**Table 1:** Baseline characteristics of randomized participants.

Variable	Narrative intervention (*n* = 90)	Active control (*n* = 90)	*p*
Age, years	10.84 ± 0.76	10.79 ± 0.81	0.672
Male/Female	46/44	43/47	0.671
Grade 5/6	48/42	45/45	0.668
Reading ability score	76.43 ± 8.25	75.91 ± 8.47	0.694
Executive-function composite	0.02 ± 0.91	−0.01 ± 0.94	0.841
RCADS total	31.42 ± 6.18	31.76 ± 6.35	0.718
Cognitive reappraisal	3.14 ± 0.62	3.11 ± 0.65	0.746

Sample size was determined before recruitment in G^*^Power 3.1 (F tests; ANOVA: repeated measures, within–between interaction). We entered a conservative small interaction effect of f = 0.10 because effects from school-based developmental interventions are heterogeneous and the study was intended to detect a modest differential trajectory rather than a large treatment effect. With α = 0.05, power (1 – β) = 0.90, two groups, four repeated measurements, an assumed correlation of 0.50 among repeated measures, and non-sphericity correction ε = 1.00, the required total sample was 179. Recruitment was therefore set at 180 so that 1:1 allocation could be maintained (90 per condition). With these inputs and *N* = 180, achieved design power was 0.903.

### 2.2 Allocation, assessor masking, and contamination safeguards

An independent researcher who had no role in recruitment, intervention delivery, or outcome assessment generated the individual-level allocation sequence by computer. Within strata defined by age (10–11 vs. 12 years), sex, and baseline reading-ability category (below vs. at/above the sample median), random permuted blocks of variable size (4 and 6) maintained 1:1 allocation while limiting predictability. Recruiters and assessors could not access the password-protected allocation file. A research administrator independent of enrolment prepared sequentially numbered, opaque, sealed envelopes in advance; each envelope contained only the assigned study condition. Envelopes were opened only after eligibility had been confirmed and T0 assessment was complete, so the enrolling researcher and baseline assessors did not know the upcoming assignment beforehand. Outcome assessors remained blinded at later assessments. Participants and instructors could not be blinded because the classroom procedures were behaviorally distinct.

To minimize contamination, intervention and active-control activities were delivered on separate timetables and, where feasible, by condition-specific instructors. Instructors were explicitly instructed not to introduce cognitive-poetics prompts, perspective-shifting exercises, or reappraisal prompts in the active-control condition. Session materials were stored separately by condition, and allocation information was not included on assessment forms used by blinded outcome assessors. No cross-condition switching occurred after randomization.

### 2.3 Intervention materials and delivery

The intervention lasted 12 weeks and comprised 36 sessions (three 40-min sessions per week). Both groups were exposed to British and American coming-of-age literary material for an equivalent amount of time. The experimental difference was the processing structure: the intervention condition explicitly guided text-world construction, character-perspective shifts, emotional interpretation, alternative meaning generation, and cognitive reappraisal, whereas the active control emphasized conventional reading comprehension, vocabulary, plot summary, and general discussion. The overall intervention structure and assessment schedule are presented in Figure 1.

**Figure 1:**
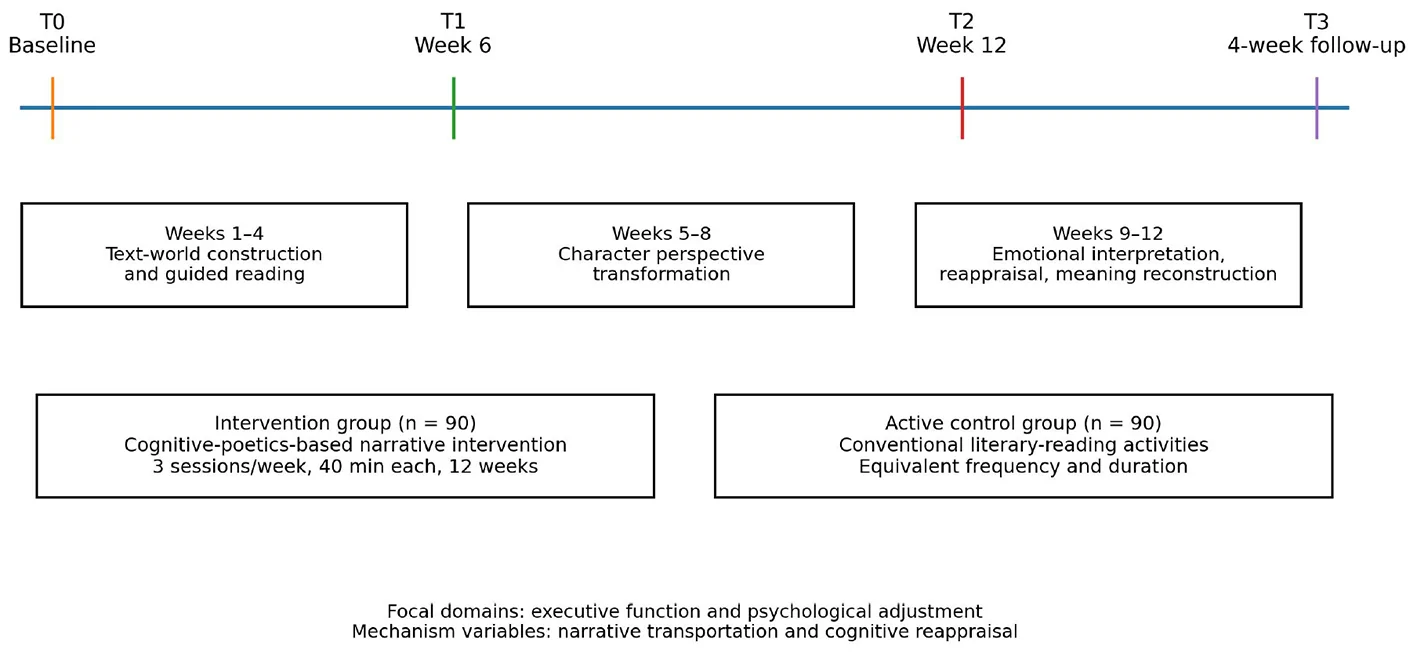
Structure and assessment schedule of the cognitive-poetics-based narrative intervention.

Texts were selected using four criteria stated in the protocol: developmental proximity of the protagonist to the target age group; relevance to growth, identity, relationships, or coping; sufficient representation of characters' thoughts and emotions; and age-appropriate emotional intensity. The material was organized into four thematic units: self-discovery/identity, interpersonal relationships/perspective understanding, setbacks/emotional adjustment, and growth/meaning reconstruction.

Novel selection was conducted before allocation by a five-member materials panel comprising two specialists in English/literature education, one developmental or educational psychologist, one primary-school literacy teacher, and one school-based counselor familiar with child safeguarding. The panel screened an initial pool of 18 British and American children's or young-adolescent novels that were potentially suitable for late-childhood readers. Candidate works were rated for protagonist-age proximity, thematic relevance to identity/relationships/coping, density of interpretable mental-state and perspective information, suitability for excerpt-based teaching, emotional intensity, and translation/cultural accessibility. Five candidates were excluded because the reading level or narrative complexity was judged unsuitable for the target age range, four because the material contained themes or emotional content judged unnecessarily mature or distressing for a universal school-based program, three because coherent excerpts could not support the planned nine-session thematic unit without extensive contextual explanation, and two because cultural/background knowledge demands would have required substantial instructor scaffolding that could confound the experimental manipulation. The remaining four works were selected by consensus because they met all core criteria and collectively provided balanced coverage of self-identity, peer/family relationships, setbacks, perspective change, and meaning reconstruction.

The intervention corpus comprised four age-appropriate British and American coming-of-age works aligned with the four thematic units: Wonder (R. J. Palacio, Alfred A. Knopf, 2012), The Boy at the Back of the Class (Onjali Q. Raúf, Orion Children's Books, 2018), Matilda (Roald Dahl, Jonathan Cape, 1988), and Holes (Louis Sachar, Farrar, Straus and Giroux, 1998). The cited English editions served as the source texts, whereas participants received standardized simplified-Chinese translations of selected excerpts rather than the English originals so that variation in English-language proficiency would not determine comprehension of the intervention materials. Selected excerpts rather than entire novels were used so that reading load was comparable across the 36 sessions. The selections emphasized protagonist perspective, interpersonal conflict, setbacks, reinterpretation, and developmental change. The literary materials and their selection rationale are summarized in Table 2.

**Table 2:** Literary materials and selection rationale.

Thematic unit	Work / author / edition	Text used and language	Selection rationale / age appropriateness
Self-discovery and identity	Wonder—R. J. Palacio; Alfred A. Knopf, 2012	Standardized simplified-Chinese research translation of selected excerpts from the cited English source edition; approximately 650–850 Chinese characters per session.	Protagonist is close to the target age range; repeated perspective shifts support identity reflection and perspective comparison.
Relationships and perspective understanding	The Boy at the Back of the Class—Onjali Q. Raúf; Orion Children's Books, 2018	Standardized simplified-Chinese research translation of selected excerpts from the cited English source edition; approximately 650–850 Chinese characters per session.	School-based peer relationships and cross-perspective understanding are developmentally proximal and suitable for guided empathy/perspective work.
Setbacks and emotional adjustment	Matilda—Roald Dahl; Jonathan Cape, 1988	Standardized simplified-Chinese research translation of selected excerpts from the cited English source edition; approximately 650–850 Chinese characters per session.	Provides clear emotional challenges and competing interpretations while remaining accessible to upper-primary readers.
Growth and meaning reconstruction	Holes—Louis Sachar; Farrar, Straus and Giroux, 1998	Standardized simplified-Chinese research translation of selected excerpts from the cited English source edition; approximately 650–850 Chinese characters per session.	Supports longitudinal reinterpretation of events, attributional change, friendship, and reconstruction of meaning across a developing plot.

For the study materials, excerpts were translated into simplified Chinese by two bilingual researchers with training in English-language/literature education. Each excerpt was translated independently, differences were reconciled against the cited English source edition, and a third bilingual reviewer checked semantic equivalence, age-appropriate wording, preservation of character perspective, and emotionally salient wording before the passage entered the intervention manual. Proper names and culture-specific references were retained where they were readily understandable; brief neutral glosses were provided only when necessary for literal comprehension and did not supply interpretive or reappraisal content. Passage length and difficulty were standardized across conditions, with typical session excerpts containing approximately 650–850 Chinese characters. The intervention and active-control groups used the same translated passage at a given session whenever possible; only the instructional prompts and processing activities differed between conditions.

Intervention sessions lasted 40 min. The sequence was fixed: 4 min to orient children to the selected passage and its prior narrative context, 10 min of guided reading, 6 min to identify characters' goals/beliefs/emotional shifts, 8 min to compare alternative character perspectives, 8 min to generate alternative interpretations or cognitive reappraisals of a difficult event, and 4 min for closing reflection. Prompts varied with the passage but followed the same functions. Examples were: “What does this character believe is happening at this point in the story?,” “How might the same event look from another character's perspective?,” “What new information could change the first interpretation?,” and “What is another way the character could understand this setback?” The discussion stayed with fictional situations and did not require personal disclosure.

Control sessions matched the intervention in literary exposure, session frequency, and the 40-min duration, but did not use the structured cognitive-poetics exercises. A typical session used 4 min for review/orientation, 16 min for reading, 8 min for vocabulary/comprehension work, 8 min for plot sequencing or factual discussion, and 4 min for summary. In this way, instructor contact and reading exposure were comparable while direct practice in perspective switching, alternative interpretation, and reappraisal was minimized.

### 2.4 Instructor preparation, intervention fidelity, and safety

Intervention delivery was standardized through a session manual, instructor training, rehearsal, and weekly implementation review. Four instructors (two assigned to each condition) had postgraduate-level training in English/literature education, applied linguistics, educational psychology, or a closely related field and at least 3 years of experience working with school-age children. None participated in outcome assessment. Before participant sessions began, all instructors completed an 8-h standardized preparation program covering the study protocol, child safeguarding, session timing, neutral response to distress, contamination prevention, and use of the condition-specific manual. Training time was matched across conditions: intervention instructors rehearsed text-world construction, perspective-shifting, alternative-interpretation, and reappraisal prompts, whereas active-control instructors rehearsed time-matched reading-comprehension, vocabulary, plot-sequencing, and factual-discussion procedures. Each instructor completed two supervised mock sessions and was required to achieve at least 85% adherence on the relevant fidelity checklist before study delivery. Brief weekly supervision meetings were then used to review timing, deviations, and condition differentiation without access to participant outcome data.

Two graduate-level research assistants who were not involved in intervention delivery or outcome testing served as fidelity observers. They completed a 4-h observation-codebook training followed by calibration on three practice recordings; independent observation began only after item-level agreement reached at least 85%. Observers necessarily could identify condition from session content, but they had no access to participants' assessment scores. Fidelity was indexed separately for intervention adherence and control-condition differentiation. Eighteen of the 72 condition-sessions (25%; nine intervention and nine control) were independently observed using a structured checklist, and eight observed sessions were double-rated. Intervention adherence averaged 92.8% (SD = 4.6; range 83.3%−100%), control-condition differentiation averaged 95.2% (SD = 3.8; range 87.5%−100%), and inter-rater agreement across double-rated sessions was ICC (2, k) = 0.91. Three minor deviations involved shortened discussion/reflection phases and were addressed during routine implementation review; no major protocol deviations were recorded.

Attendance and assessment completion were recorded across all 36 sessions. Mean attendance was 33.1 ± 2.8 sessions (91.9%) in the intervention group and 32.8 ± 3.0 sessions (91.1%) in the active-control group. A per-protocol threshold of ≥80% attendance (≥29 of 36 sessions) was used for the attendance-based sensitivity set. Children's responses to the literary material were monitored for discomfort, and no serious adverse events were reported. Because participants and instructors knew which classroom activities they received, performance and expectancy effects remain possible and are considered in the limitations.

### 2.5 Outcome and mechanism measures

Assessments were administered at T0, T1, T2, and T3 by trained assessors blinded to allocation, using the same assessment instructions across occasions. Executive function and psychological adjustment were treated as the focal outcome domains, while narrative transportation and cognitive reappraisal were mechanism measures. Because the study was not preregistered with a formal primary/secondary outcome hierarchy, the reporting hierarchy used here should not be interpreted as preregistered.

#### 2.5.1 Executive function

Inhibitory control was assessed with a computerized arrow Flanker task implemented in E-Prime 3.0. After 24 practice trials, children completed 120 scored trials (60 congruent and 60 incongruent). A fixation cross was presented for 500 ms, followed by the stimulus until response or a 1,500-ms deadline; inter-trial intervals varied randomly from 800 to 1,200 ms. Error trials, omissions, responses < 200 ms, and responses exceeding 1,500 ms were excluded from reaction-time calculations. The domain score was the mean of baseline-referenced z(accuracy) and z(–reaction time), so higher scores indicated better inhibitory control. Split-half reliability was 0.84, 0.86, 0.86, and 0.85 at T0–T3, respectively. Working memory was assessed with a computerized backward digit-span task. Four practice trials were followed by two scored trials at each span length from 2 to 8 digits (maximum 14 scored trials); digits were shown for 1,000 ms each with a 250-ms inter-digit interval. The working-memory score averaged baseline-referenced z (proportion of correctly reproduced sequences) and z(maximum span); split-half reliability was 0.81, 0.82, 0.84, and 0.82 across T0–T3. Cognitive flexibility was assessed with a computerized color–shape task-switching paradigm comprising 24 practice trials and 128 scored mixed-block trials (64 repeat and 64 switch trials). A 500-ms task cue preceded a stimulus with a 2,000-ms response deadline; errors, omissions, and responses < 200 ms were excluded from RT analyses. The flexibility score averaged baseline-referenced z (switch accuracy) and z (–RT switch cost), where switch cost equaled mean switch-trial RT minus mean repeat-trial RT. Split-half reliability was 0.82, 0.84, 0.85, and 0.83 at T0–T3. All three domain scores at every occasion were standardized against the pooled T0 mean and SD rather than re-standardized separately at each wave. The executive-function composite was the arithmetic mean of the three oriented domain z scores.

#### 2.5.2 RCADS

Anxiety and depressive symptoms were assessed using the 47-item child-report Revised Child Anxiety and Depression Scale (RCADS-47; Chorpita et al., 2000). Items were rated from 0 (never) to 3 (always), and the raw total anxiety-and-depression score was analyzed; higher scores indicate greater symptom burden. Scale scores were prorated only when item-level missingness remained within the instrument's allowable scoring range. In the present sample, Cronbach's α/McDonald's ω were 0.93/0.94 at T0, 0.92/0.93 at T1, 0.94/0.94 at T2, and 0.93/0.94 at T3. In this non-clinical school sample, RCADS scores are interpreted as symptom severity rather than diagnosis.

#### 2.5.3 Strengths and difficulties questionnaire

Behavioral and emotional adjustment was assessed with the Strengths and Difficulties Questionnaire (SDQ; Goodman, 1997), a 25-item measure covering emotional symptoms, conduct problems, hyperactivity/inattention, peer problems, and prosocial behavior. Parent and teacher versions were collected. For each informant, the four difficulties subscales were summed on the original 0–40 scale. When both parent and teacher reports were valid, the two raw total-difficulties scores were averaged to form the multi-informant SDQ difficulties score; when only one valid informant report was available, the available informant score was retained. Parent-only and teacher-only scores were examined as sensitivity outcomes. Internal consistency for the parent difficulties score was α = 0.82, 0.83, 0.84, and 0.83 across T0–T3; corresponding teacher α values were 0.85, 0.86, 0.87, and 0.86. Parent–teacher agreement (ICC) was 0.62, 0.65, 0.68, and 0.66 across T0–T3.

#### 2.5.4 Emotion regulation and cognitive reappraisal

Self-reported cognitive reappraisal was assessed using the Emotion Regulation Questionnaire for Children and Adolescents (ERQ-CA; Gullone and Taffe, 2012). The six-item cognitive-reappraisal subscale was rated on a 1–5 response scale and scored as the item mean, with higher scores indicating more frequent reinterpretation as an emotion-regulation strategy. In the present sample, α/ω = 0.84/0.85 at T0, 0.86/0.87 at T1, 0.87/0.88 at T2, and 0.86/0.87 at T3.

As a performance-based complement to habitual self-report, children also completed a scenario-based reappraisal task under standardized conditions. The task used six short, age-relevant vignettes: two academic-setback scenarios (e.g., receiving an unexpectedly poor result or making an error during a classroom task), two peer-interaction scenarios (e.g., exclusion or an ambiguous negative comment), and two performance/criticism scenarios (e.g., unsuccessful performance followed by corrective feedback). Each vignette was 60–90 words long and followed the same presentation format without asking children to disclose personal experiences. After reading a vignette, children responded to two prompts: (1) “What is another way to understand what happened?” and (2) “What could the child do or think next to feel or cope better?” Depending on the assessment format used at that occasion, responses were either entered in writing or given orally and transcribed verbatim.

Scoring used two prespecified 0–2 dimensions for each scenario. For reinterpretation quality, 0 indicated no alternative interpretation or repetition of the negative appraisal, 1 indicated a plausible but relatively superficial alternative construal, and 2 indicated a clear, context-sensitive reinterpretation that reduced threat, broadened causal explanation, or introduced a constructive alternative meaning. Response flexibility was scored 0 for no usable response or a clearly avoidant/aggressive response, 1 for one feasible constructive response, and 2 for a flexible or contingent response that identified more than one adaptive option or linked action to the revised interpretation. The two dimension scores were added within each vignette (0–4) and then across the six vignettes, producing a total performance score from 0 to 24; higher values indicated stronger demonstrated reappraisal ability. Two trained raters who were blinded to treatment assignment and assessment scores independently scored a randomly selected 25% of protocols. Reliability was estimated before disagreements were resolved by discussion, and the double-scored subset yielded ICC (2, k) = 0.88. The scenario-based score remained a supplementary performance measure and was not included in the longitudinal mechanism model. The Mplus analysis used the ERQ-CA cognitive-reappraisal score so that one mediator was measured consistently across waves.

#### 2.5.5 Narrative transportation

Narrative transportation was assessed as a candidate mechanism using an 11-item child-adapted version of the general Narrative Transportation Scale derived from Green and Brock (2000), excluding story-specific character items. Items were rated from 1 (not at all) to 7 (very much) and averaged, with higher scores indicating stronger cognitive, emotional, and imagery-based immersion. Transportation was assessed at T1 and T2; the longitudinal indirect-effect model used T1 transportation to preserve temporal ordering. At T1, the intervention and control means were 4.92 ± 0.82 and 4.31 ± 0.86, respectively; α = 0.88 and ω = 0.89. T2 descriptive means were 5.17 ± 0.79 and 4.39 ± 0.84.

#### 2.6 Statistical analysis

Descriptive statistics were calculated for all outcomes at each available assessment point. Baseline group comparisons were summarized using standardized or conventional descriptive comparisons rather than using non-significant baseline *p* values as proof that randomization “worked.” Randomization was evaluated primarily by the allocation procedure and the magnitude of baseline imbalance.

Longitudinal group differences were estimated with restricted-maximum-likelihood (REML) linear mixed-effects models. Fixed effects were group, assessment time as a four-level categorical factor, and group × time. A participant-level random intercept represented stable between-child differences, while a first-order autoregressive [AR(1)] residual covariance structure represented within-child correlation from T0 to T3. Denominator degrees of freedom used the Satterthwaite approximation. Covariates were not selected from baseline *p* values in the primary models; age, sex, and baseline reading ability were entered together only in sensitivity analyses. Differential change was evaluated with the group × time interaction. We checked assumptions with residual and Q–Q plots, standardized residuals and influential cases, and the pattern of residual variance across groups and assessment occasions.

All 180 randomized participants remained in the intention-to-treat (ITT) analysis according to their assigned group. REML used every available repeated observation under a missing-at-random assumption, without single-value imputation. Assessment completion was 180 at T0, 175 at T1 (88 intervention, 87 control), 170 at T2 (86 intervention, 84 control), and 164 at T3 (83 intervention, 81 control). The attendance-based per-protocol sensitivity set required ≥80% attendance (≥29/36 sessions) and included 169 children (85 intervention, 84 control). Reasons for non-completion were recorded at each assessment and are reported in Section 3.1. Missing assessments were not replaced by single-value imputation.

For effect-size interpretation, model-level partial η^2^ values are reported alongside unadjusted between-group standardized mean differences calculated directly from the displayed means and SDs. For a given assessment, Cohen's d was calculated as the difference in group means divided by the pooled within-group SD; 95% CIs were calculated using the standard sampling variance of d. These descriptive d values complement, but do not replace, the longitudinal interaction effect.

The executive-function composite, RCADS total score, and SDQ difficulties score were treated as focal domain-level outcomes. Component-level executive-function analyses, cognitive reappraisal, and other supplementary contrasts were treated as secondary/exploratory. No multiplicity adjustment was applied to these exploratory analyses; therefore, effect estimates and confidence intervals are emphasized and secondary *p* values are interpreted cautiously. Because the study was not preregistered, this hierarchy should be understood as the reporting hierarchy used in the present analysis rather than as a preregistered outcome hierarchy. Changes in psychological adjustment indicators across assessment points are displayed in Figure 4.

Indirect effects were estimated in Mplus 8.8 using maximum-likelihood estimation, full-information maximum likelihood for incomplete endogenous-variable data, and 5,000 non-parametric bootstrap resamples for confidence intervals. Treatment was coded 0 = active control and 1 = narrative intervention. The temporally ordered model specified intervention assignment → T1 narrative transportation → T2 cognitive reappraisal → T3 outcome, with T0 cognitive reappraisal and the corresponding T0 outcome entered as baseline controls. In addition to the serial pathway, direct intervention → T2 reappraisal and T1 transportation → T3 outcome paths were estimated, allowing decomposition of serial-specific, parallel-specific, total indirect, direct, and total intervention effects. Standardized (STDYX) coefficients are reported for individual paths and effect decompositions; bootstrap confidence intervals were used for indirect, direct, and total effects. Age, sex, and baseline reading ability were examined in sensitivity models rather than selected *post hoc*.

Model fit for the longitudinal path model was evaluated using the model χ^2^ test together with CFI, TLI, RMSEA and its 90% confidence interval, and SRMR. Because χ^2^ is sensitive to sample size, interpretation emphasized the pattern across indices rather than a single cutoff; values near or above 0.95 for CFI/TLI and near or below 0.06–0.08 for RMSEA/SRMR were treated as indicating acceptable-to-good approximate fit. The fitted model showed χ^2^ (14) = 18.62, p = 0.180, CFI = 0.979, TLI = 0.951, RMSEA = 0.043 (90% CI [.000, 0.089]), and SRMR = 0.034.

All statistical tests were two-sided with α = 0.05. Analyses were conducted in SPSS 29.0 and Mplus 8.8.

## 3 Results

### 3.1 Participant flow and baseline characteristics

Of 214 children screened, 34 were not randomized (21 did not meet eligibility criteria and 13 declined participation), leaving 180 randomized participants (90 per condition). T1 was completed by 175 children (88 intervention, 87 control). The five T1 non-completions comprised two intervention participants (one acute illness and one school-related scheduling conflict) and three control participants (one acute illness and two family/school scheduling conflicts). By T2, an additional five children were no longer available for assessment, yielding 170 completers (86 intervention, 84 control): in the intervention group, one child had transferred/relocated and one family withdrew because of competing time demands; in the control group, one child relocated, one family withdrew because of competing extracurricular commitments, and one participant could not be scheduled after repeated absence. Between T2 and the 4-week T3 follow-up, three further participants in each group did not complete assessment, yielding 164 completers (83 intervention, 81 control). The additional intervention-group losses comprised one relocation and two participants unavailable within the follow-up assessment window; the additional control-group losses comprised one relocation, one family withdrawal from follow-up, and one participant unavailable after repeated contact attempts. No recorded non-completion was attributed to a serious adverse event. The ITT mixed-model analyses retained all 180 randomized participants using all available repeated observations. The attendance-based per-protocol set comprised 169 children (85 intervention, 84 control). Participant recruitment, allocation, and follow-up procedures are illustrated in Figure 2.

**Figure 2:**
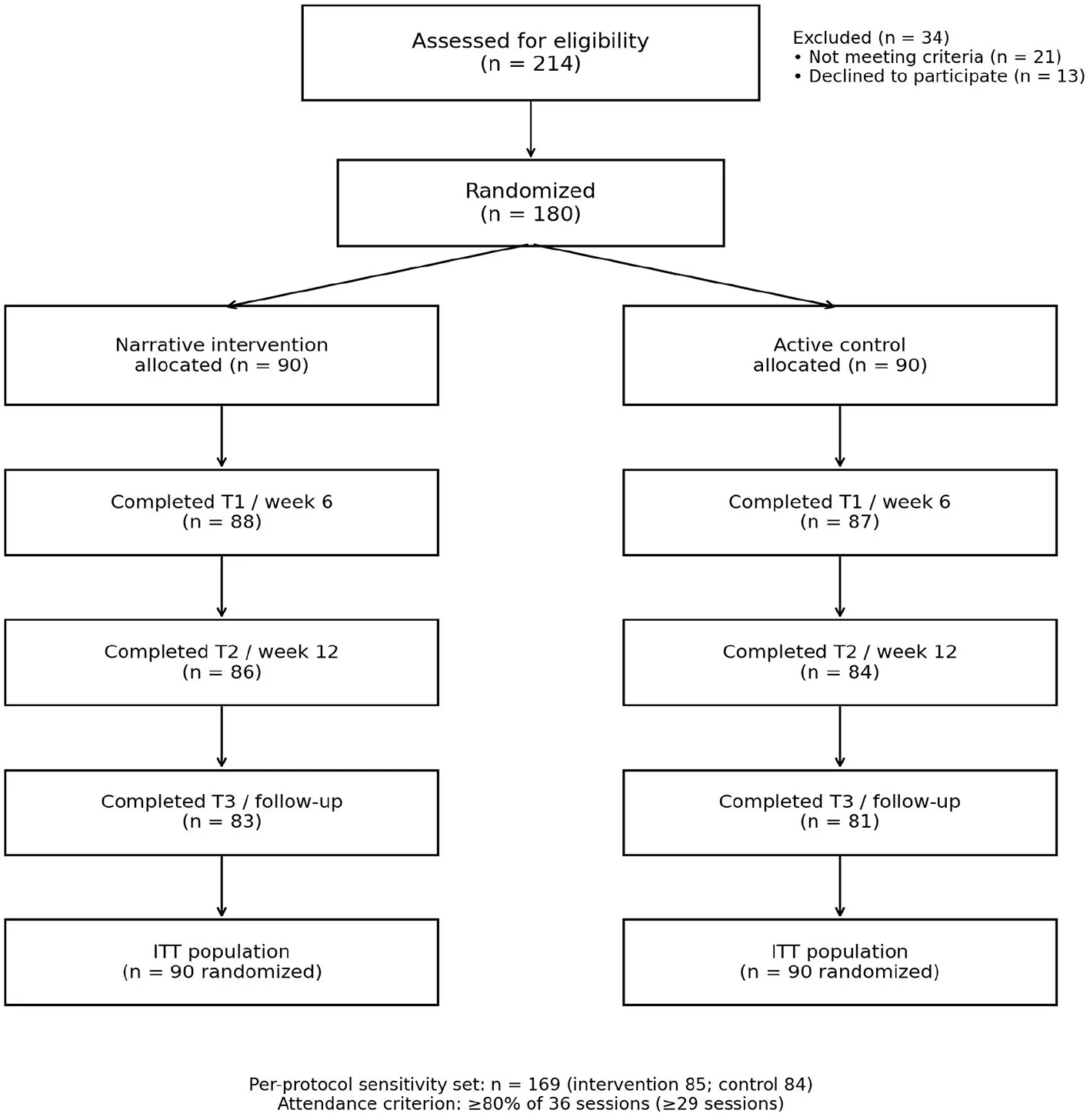
Participant flow and the distinction between the intended ITT population and the attendance-based per-protocol set.

The randomized groups were descriptively similar at baseline. Because significance tests after randomization do not establish whether randomization succeeded, the *p* values are provided for description rather than as a validity check; the allocation procedure and the size of any imbalance are more informative.

### 3.2 Intervention exposure and fidelity

Attendance was high in both conditions: 33.1 ± 2.8 of 36 sessions (91.9%) in the intervention group and 32.8 ± 3.0 sessions (91.1%) in the active control. Eighteen condition-sessions were independently observed (nine per condition). Intervention adherence averaged 92.8% (SD = 4.6; range 83.3%−100%), and control-condition differentiation averaged 95.2% (SD = 3.8; range 87.5%−100%). Eight observed sessions were double-rated, yielding ICC (2, k) = 0.91. Three minor deviations involved shortened discussion/reflection phases; no major protocol deviations or serious adverse events were recorded.

### 3.3 Executive-function outcomes

Executive-function trajectories differed across groups over time (group × time: F = 18.64, *p* < 0.001, partial η^2^ = 0.095). The intervention mean rose from 0.02 ± 0.91 at T0 to 0.51 ± 0.86 at T2; the active-control mean changed from −0.01 ± 0.94 to 0.18 ± 0.89 over the same period. At T3, the corresponding means were 0.46 ± 0.84 and 0.21 ± 0.87. The executive-function trajectories across assessment points are shown in Figure 3.

**Figure 3:**
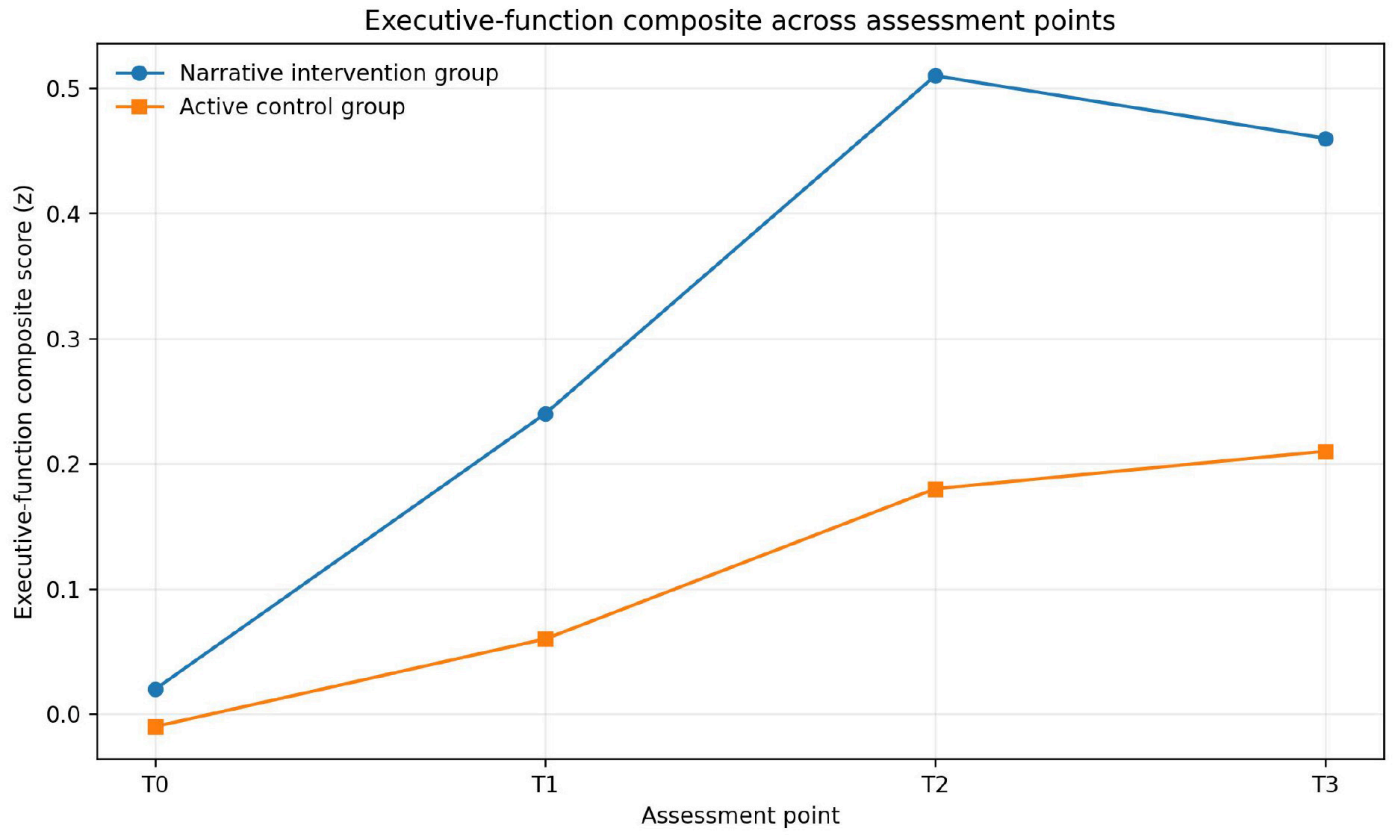
Executive-function composite scores across T0, T1, T2, and T3.

At T2, the unadjusted between-group standardized mean difference for the executive-function composite was d = 0.38 (95% CI [0.07, 0.68]), calculated from the displayed summary statistics and completion counts (86 intervention, 84 control). This effect size describes the post-intervention contrast; the longitudinal group × time test remains the primary test of differential change. The corresponding descriptive values are summarized in Table 3. The relevant tabulated materials are provided in Tables 1 and 3.

**Table 3:** Executive-function composite across assessment points.

Assessment	Narrative intervention	Active control	Unadjusted between-group d (95% CI)
T0	0.02 ± 0.91	−0.01 ± 0.94	0.03 [−0.26, 0.32]
T1	0.24 ± 0.88	0.06 ± 0.90	0.20 [−0.09, 0.50]
T2	0.51 ± 0.86	0.18 ± 0.89	0.38 [0.07, 0.68]
T3	0.46 ± 0.84	0.21 ± 0.87	0.29 [−0.02, 0.60]

The component scores followed the same broad direction as the composite, although the trajectories were not identical. Baseline-referenced means for the intervention/control groups were 0.01/−0.03, 0.27/0.08, 0.58/0.22, and 0.52/0.24 for inhibitory control at T0–T3; 0.03/0.00, 0.20/0.04, 0.44/0.15, and 0.39/0.19 for working memory; and 0.02/0.00, 0.25/0.06, 0.51/0.17, and 0.47/0.20 for cognitive flexibility.

### 3.4 Psychological-adjustment outcomes

RCADS total score and SDQ difficulties also showed group-dependent change over time (RCADS: F = 15.72, *p* < 0.001, partial η^2^ = 0.086; SDQ: F = 12.48, *p* < 0.001, partial η^2^ = 0.074). At T2, children in the intervention group had lower RCADS and SDQ difficulties scores and higher reappraisal scores than those in the active control. These are symptom and adjustment indicators in a non-clinical sample; they should not be read as evidence that psychiatric disorders were treated. Changes in psychological adjustment indicators across assessment points are displayed in Figure 4.

**Figure 4:**
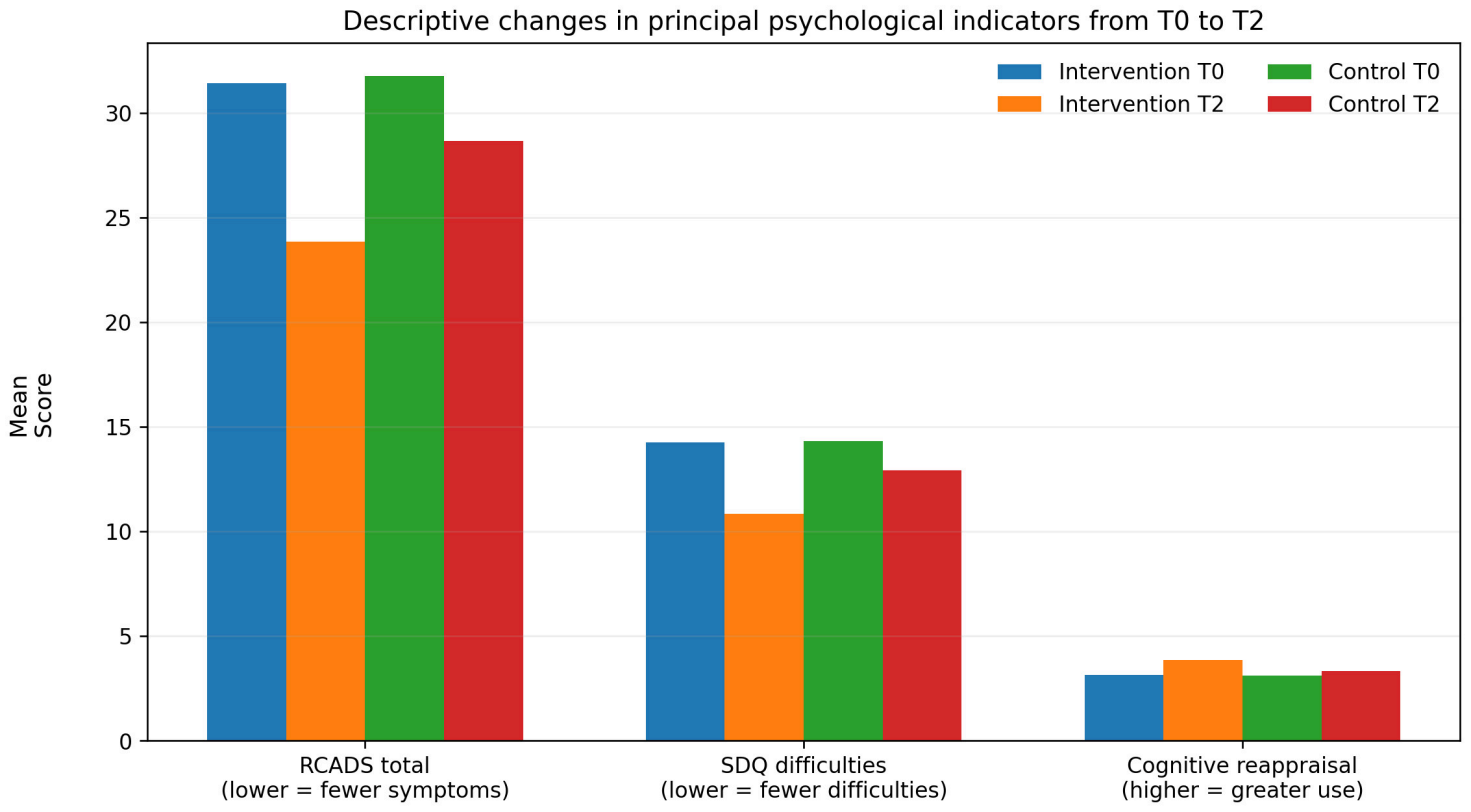
Descriptive changes from T0 to T2 in RCADS total, SDQ difficulties, and cognitive reappraisal.

The descriptive sample sizes were 90/90 at T0, 88/87 at T1, 86/84 at T2, and 83/81 at T3 for the intervention/control groups. Table 4 includes each planned assessment point so that the post-intervention and short-term follow-up patterns can be read directly.

**Table 4:** Psychological-adjustment and reappraisal measures across assessment points.

Outcome	Time	Narrative intervention	Active control	Unadjusted d (95% CI)
RCADS total	T0	31.42 ± 6.18	31.76 ± 6.35	−0.05 [−0.35, 0.24]
	T1	27.68 ± 5.96	30.25 ± 6.18	−0.42 [−0.72, −0.12]
	T2	23.84 ± 5.72	28.67 ± 6.01	−0.82 [−1.14, −0.51]
	T3	24.56 ± 5.91	28.21 ± 6.18	−0.60 [−0.92, −0.29]
SDQ difficulties	T0	14.26 ± 3.42	14.31 ± 3.51	−0.01 [−0.31, 0.28]
	T1	12.61 ± 3.28	13.69 ± 3.41	−0.32 [−0.62, −0.02]
	T2	10.83 ± 3.11	12.92 ± 3.35	−0.65 [−0.96, −0.34]
	T3	11.18 ± 3.20	12.76 ± 3.39	−0.48 [−0.79, −0.17]
Cognitive reappraisal	T0	3.14 ± 0.62	3.11 ± 0.65	0.05 [−0.25, 0.34]
	T1	3.50 ± 0.65	3.20 ± 0.64	0.47 [0.16, 0.77]
	T2	3.87 ± 0.68	3.34 ± 0.63	0.81 [0.50, 1.12]
	T3	3.79 ± 0.70	3.31 ± 0.66	0.71 [0.39, 1.02]

### 3.5 Indirect-effect model

In the temporally ordered structural model, intervention assignment was associated with higher T1 narrative transportation (standardized β = 0.42, *p* < 0.001). T1 transportation predicted higher T2 cognitive reappraisal (β = 0.36, *p* < 0.001), and intervention assignment also had a direct path to T2 reappraisal (β = 0.22, *p* = 0.004). T2 reappraisal predicted higher T3 executive-function scores (β = 0.28, *p* < 0.001) and lower T3 psychological-difficulty scores (β = −0.31, *p* < 0.001). T1 transportation additionally showed a small path to T3 executive function (β = 0.09, *p* = 0.164) and a negative path to T3 psychological difficulties (β = −0.16, *p* = 0.031). The serial-specific indirect effect through T1 transportation and T2 reappraisal was β = 0.04 (95% bootstrap CI [.01, 0.08]) for executive function and β = −0.05 (95% CI [−0.10, −0.02]) for psychological difficulties. After inclusion of the parallel indirect routes through transportation and reappraisal, the total indirect effects were β = 0.14 (95% CI [.06, 0.25]) and β = −0.18 (95% CI [−0.31, −0.09]), respectively. For executive function, the residual direct intervention effect was β = 0.13 (95% CI [.02, 0.25], *p* = 0.029) and the total intervention effect was β = 0.27 (95% CI [.13, 0.41], *p* < 0.001). For psychological difficulties, the residual direct intervention effect was β = −0.24 (95% CI [−0.39, −0.10], *p* = 0.001) and the total intervention effect was β = −0.42 (95% CI [−0.58, −0.27], *p* < 0.001). Thus, the point estimates satisfy the expected decomposition of total effect into direct plus total indirect effect for each outcome.

Separating T1 transportation, T2 reappraisal, and T3 outcomes provides a clearer temporal sequence than a same-wave mediation model, and the analysis controlled baseline reappraisal and outcome values. The indirect effects should still be read as model-based associations. Mediator levels were not independently randomized, and other unmeasured processes could have produced part of the observed pathway. The proposed indirect-effect model is displayed in Figures 1–5.

**Figure 5:**
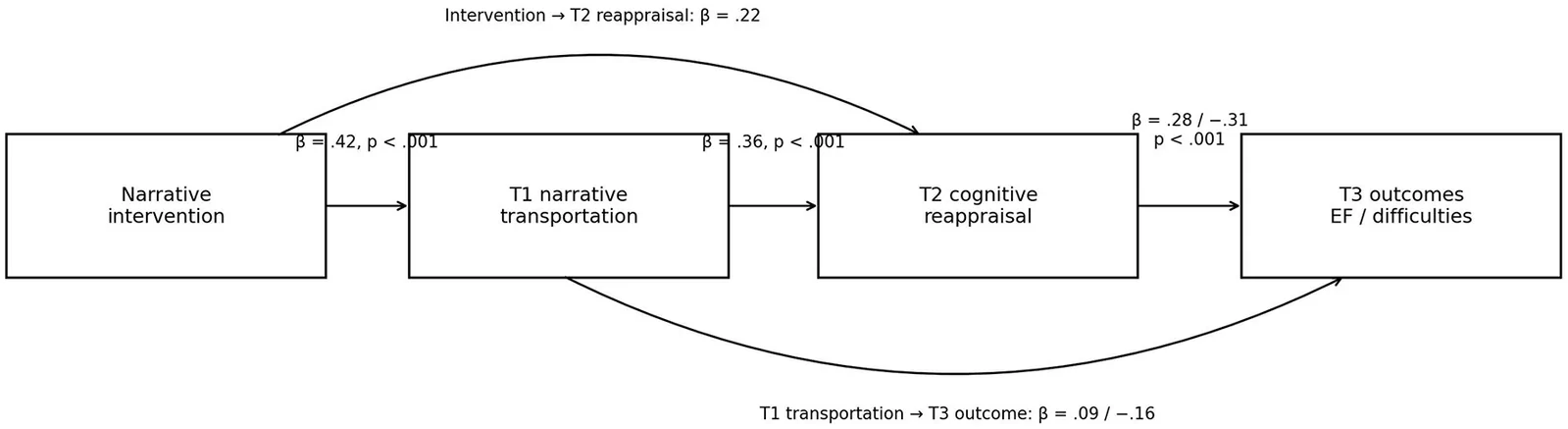
Temporally ordered indirect-effect model: intervention assignment → T1 narrative transportation → T2 cognitive reappraisal → T3 executive-function/psychological-adjustment outcome. Baseline reappraisal and the corresponding T0 outcome were controlled. Standardized serial-specific indirect effects were 0.04 and −0.05; the corresponding total indirect effects were 0.14 and −0.18.

The fitted model yielded χ^2^(14) = 18.62, *p* = 0.180, CFI = 0.979, TLI = 0.951, RMSEA = 0.043 (90% CI [.000, 0.089]), and SRMR = 0.034. This pattern is compatible with acceptable-to-good global fit. Fit statistics, however, cannot show that the proposed mechanism is either unique or causal.

## 4 Discussion

### 4.1 Interpreting the executive-function pattern

The two groups did not follow the same executive-function trajectory. Because both conditions involved literary reading over the same broad period and included instructor contact, the comparison is more specific than reading vs. no treatment. The intervention differed in what children did with the text: they shifted perspectives, retrieved earlier narrative information, compared alternative interpretations, and worked through reappraisal prompts. These elements were delivered together. The present data therefore cannot isolate the contribution of any one component.

One reason the pattern is theoretically plausible is that narrative interpretation places several demands on executive control. A reader may have to hold a character's goals and earlier events in working memory, suppress an initial interpretation after new information appears, and move between competing viewpoints. That resembles the executive domains described by Miyake et al. (2000) and Diamond (2013). Similarity in task demands, however, is not evidence of a specific training mechanism. Repeated testing may have produced practice gains, and greater classroom engagement could also have contributed to the observed difference.

The follow-up pattern was less clear-cut. The descriptive executive-function gap at T3 was smaller than at T2, and the unadjusted between-group d confidence interval included zero. We therefore interpret the result as partial short-term persistence rather than a sustained developmental benefit. Whether the difference lasts beyond the immediate post-program period remains an open question.

### 4.2 Psychological adjustment in relation to previous narrative and school-based work

Psychological-adjustment measures moved in the same favorable direction: the intervention group had lower RCADS and SDQ difficulty scores at post-intervention. This result fits a broader literature showing that structured school-based social-emotional activities can affect adjustment (Durlak et al., 2011; Taylor et al., 2017), but the closest comparison is the smaller literature on bibliotherapy and narrative work with children. Such programs have been used to support social-emotional skills and resilience, while more clinically oriented narrative interventions have targeted selected anxiety and social-emotional problems (Beaudoin et al., 2016; Veronese and Barola, 2018; Zarra-Nezhad et al., 2023; Peker et al., 2026).

That literature also marks an important boundary around the present study. Our program was not a clinical narrative-therapy protocol; fictional coming-of-age texts were the shared material, and the sessions were organized as a developmental educational activity. Redman et al. (2026) found encouraging but heterogeneous results in school-based creative bibliotherapy and noted substantial risk-of-bias concerns in many studies. Using an attention-matched active control addresses one of those problems, but it does not remove expectancy, instructor, or peer-discussion effects.

The size of the post-intervention difference also depended on the measure. The displayed means show larger standardized contrasts for RCADS and reappraisal than for the executive-function composite. That uneven pattern is difficult to reconcile with the idea of one broad “therapeutic” effect. It is more consistent with domain-specific changes in symptoms, adjustment, and cognitive-emotional processing within a non-clinical sample.

### 4.3 Narrative transportation and cognitive reappraisal as candidate mechanisms

The proposed pathway rests on two linked processes. Transportation matters because an absorbed reader attends closely to a character's perspective and to the consequences of unfolding events (Green and Brock, 2000). Reappraisal offers a different link: it requires generating another meaning for an emotionally significant situation, a process that could connect guided narrative work with later emotional adjustment (Gross, 1998; Gross and John, 2003). The mechanism model tested these processes in sequence rather than treating them as a single construct.

The measurements were spaced so that T1 transportation came before T2 reappraisal, and T2 reappraisal came before the T3 outcomes; baseline reappraisal and the corresponding baseline outcome were controlled. The model also estimated the serial-specific indirect path, the broader total indirect effect, and the remaining direct intervention effect separately. A direct association persisted for both outcome domains after the indirect paths were included, meaning that transportation and reappraisal accounted for only part of the intervention–outcome relation. This improves on a same-wave analysis, but it is not a causal mediation test. The mediators were not randomized, and unmeasured time-varying influences may still contribute.

Several other processes could have contributed to the same pattern. Children may have become more engaged with reading, felt more supported by instructors, taken part in richer peer discussion, or simply elaborated their answers more often. Program expectations are another possibility. Any of these factors could covary with transportation and adjustment without belonging to the proposed serial pathway. Future studies should measure such alternatives directly and, where feasible, manipulate individual intervention components.

### 4.4 Implications for educational practice

The educational implication is narrower than turning literary reading into therapy. In an ordinary reading lesson, children can be asked not only what happened, but also to defend competing interpretations, compare viewpoints, trace shifts in a character's goals or beliefs, and propose more than one response to a setback. Those activities remain part of literacy instruction while also engaging cognitive and social-emotional processing.

Implementation matters. In the 2026 review of school bibliotherapy, favorable studies repeatedly used structured interaction, age-appropriate text selection, and trained delivery (Redman et al., 2026). A workable program therefore depends on instructors who understand the procedure, prompts that are specified in advance, fidelity criteria, and a plan for responding to emotionally difficult material. These safeguards are especially relevant when the texts come from a cultural or linguistic setting different from that of the participating children.

## 5 Limitations and future directions

A 4-week follow-up can show short-term persistence, but it cannot establish long-term developmental maintenance. Blinding was also incomplete: outcome assessors were blinded, whereas participants and instructors knew which classroom activities they received, leaving scope for expectancy and performance effects. Sessions took place in educational groups, so children sharing an instructor or session may have produced partially correlated responses. Instructor/session clustering was not modeled. Future analyses should include a group or instructor random effect when identifiers are available and the number of clusters is sufficient.

The measurement strategy brings another set of limitations. Psychological adjustment relied partly on child, parent, and teacher reports. When both informants provided valid SDQ difficulties scores, the parent and teacher values were averaged on the original 0–40 scale; if only one report was usable, that informant's score was retained. Parent–teacher agreement was moderate, which means that informant-specific perspectives and discrepancies remain relevant. Repeated executive-function testing adds a different uncertainty. Practice effects may have occurred in both groups, although the active control makes a simple retest explanation less likely.

The use of British and American source texts in standardized simplified-Chinese translation adds a cultural and linguistic limitation. Translation reduced confounding by English-language proficiency, but it can also change character voice, emotional nuance, and the emphasis placed on particular perspectives. Neutral glosses were restricted to literal comprehension; even so, ideas about family relationships, independence, school experience, or emotional expression may not carry exactly the same meaning across cultural settings. Direct comparison of culturally local and cross-cultural materials, together with formal evaluation of source-language and translated versions, would help clarify this issue.

Cognitive reappraisal occupies two roles in the study: it is a developmental outcome of interest and the second mediator in the mechanism model. Using T2 reappraisal to predict T3 outcomes while controlling T0 reappraisal and baseline outcomes reduces the immediate overlap, but does not remove it. Future studies would benefit from additional performance-based measures and from designs that manipulate candidate mechanisms rather than only observe their change.

The indirect-effect model therefore needs cautious interpretation even with T1 → T2 → T3 ordering. Alternative pathways and unmeasured time-varying confounding cannot be ruled out. The study was also not preregistered with a formal primary/secondary outcome hierarchy, so exploratory secondary *p* values should not be treated as if they came from a prespecified confirmatory family. Finally, the recruitment settings limit how far these findings can be generalized. Replication in other regions, socioeconomic backgrounds, languages, and educational systems is needed before extending the conclusions more broadly.

## 6 Conclusion

Across the 12-week program, executive-function performance and several indicators of psychological adjustment changed more favorably in children assigned to the cognitive-poetics-based narrative intervention than in those assigned to the active literary-reading control. This should not be interpreted as evidence for clinical “narrative therapy.” The narrower finding is that the way children are guided to work with a story may matter beyond exposure to reading alone. The post-intervention differences and the short follow-up warrant further testing, while stronger claims about causal mediation or durable developmental benefit require longer follow-up and designs focused more directly on mechanism.

## Data Availability

The raw data supporting the conclusions of this article will be made available by the authors, without undue reservation.
